# Energy and Exergy Analyses of a Solid Oxide Fuel Cell-Gas Turbine-Organic Rankine Cycle Power Plant with Liquefied Natural Gas as Heat Sink

**DOI:** 10.3390/e20070484

**Published:** 2018-06-22

**Authors:** Mohammad H. Ahmadi, Mirhadi S. Sadaghiani, Fathollah Pourfayaz, Mahyar Ghazvini, Omid Mahian, Mehdi Mehrpooya, Somchai Wongwises

**Affiliations:** 1 Faculty of Mechanical Engineering, Shahrood University of Technology, Shahrood 3619995161, Iran; 2 Renewable Energies and Environment Department, Faculty of New Sciences and Technologies, University of Tehran, Tehran 1417466191, Iran; 3 Fluid Mechanics, Thermal Engineering and Multiphase Flow Research Lab. (FUTURE), Department of Mechanical Engineering, Faculty of Engineering, King Mongkut’s University of Technology Thonburi, Bangmod, Bangkok 10140, Thailand; 4 School of Aeronautic Science and Engineering, Beihang University, Beijing 100191, China; 5 Hydrogen and Fuel Cell Laboratory, Faculty of New Sciences and Technologies, University of Tehran, Tehran 14176-14418, Iran

**Keywords:** organic Rankine cycle, LNG, solid oxide fuel cell, exergy efficiency, exergy destruction

## Abstract

An exergy analysis of a novel integrated power system is represented in this study. A Solid Oxide Fuel Cell (SOFC), which has been assisted with a Gas Turbine (GT) and Organic Rankine Cycle (ORC) by employing liquefied natural gas (LNG) as a heat sink in a combined power system is simulated and investigated. Initially in this paper, the integrated power system and the primary concepts of the simulation are described. Subsequently, results of the simulation, exergy analysis, and composite curves of heat exchangers are represented and discussed. The equations of the exergy efficiency and destruction for the main cycle’s units such as compressors, expanders, pumps, evaporators, condensers, reformers, and reactors are presented. According to the results, the highest exergy destruction is contributed to the SOFC reactor, despite its acceptable exergy efficiency which is equal to 75.7%. Moreover, the exergy efficiencies of the ORC cycle and the whole plant are determined to be 64.9% and 39.9%, respectively. It is worth noting that the rational efficiency of the integrated power system is 53.5%. Among all units, the exergy efficiency of the LNG pump is determined to be 11.7% the lowest exergy efficiency among the other investigated components, indicating a great potential for improvements.

## 1. Introduction

Owing to the global environmental concerns and energy crisis, applications of renewable energy, for instance, wind/solar energy, merit high attention [1,2,3]. Whereas the artificial refrigerants show significantly high define (GWP) and define (ODP), recently the environmentally friendly ecological refrigerant carbon dioxide (CO_2_) has achieved significant attention and has been recognized as an appropriate substitute refrigerant due to its outstanding heat transfer features, and its non-toxicity and non-flammability [4,5]. Therefore, machinery systems employing CO_2_ as working fluid to transform absorbed heat into thermal, mechanical energy and hereafter electricity probably represent the greatest advances among other current prospects. Medium heat transfer fluids are extensively utilized in various energy systems for transferring heat between sub-systems. Nowadays, several scholars have concentrated on refining the performances of the solar system, particularly solar collectors’ effect, and other elements on the thermal cycle performances. For instance, variables affecting the operation of water-in-glass collector tubes have been examined by Morrison and Budihardjo. Additionally, the performance of a water-in-glass evacuated tube solar heater has been studied. Riffat and Zhao [6,7] studied the heat transfer and thermodynamic features of a hybrid heat pipe solar collector/CHP system employing *n*-pentane as working fluid and the system worked on a representative Rankine cycle.

Additionally, working fluids properties affect the thermodynamic performance of cycles. Applying appropriate working fluid in a cycle can lead to higher efficiency or output power. One of the methods which are applicable is utilizing transcritical cycles to achieve better performance [8]. Several studies have focused on transcritical cycles operating with CO_2_ as working fluid. An analysis of the transcritical CO_2_ refrigeration cycle associated with the second law has been demonstrated by Fartaj et al. [9]. Yang et al. [10] carried out an exergy analysis on the transcritical CO_2_ refrigeration cycle working with an expander. Chen et al. [9] and Cayer et al. [11] implemented a comprehensive investigation of the CO_2_ Rankine cycle. These types of research focused on analyzing the system variables on a particular condition. There are limited investigations concentrating on the system performance with respect to time, not to declare the CO_2_ Rankine cycle exergy analysis with time.

Furthermore, global warming and air pollution which lead to environmental issues are growing due to industries development and daily rise in energy demand. Natural gas (NG) has been known as an eco-friendly type of energy source which could be widely utilized in the combustion process [12]. Consequently, using NG as an alternative resource for the sake of meeting the growing energy demand and reducing the environmental pollution is considered as a proper approach. Liquefied natural gas (LNG) is natural gas which has been converted into a liquid form in order to be transportable for long distances with lower difficulty [13]. Moreover, through the liquefying procedure, LNG can deliver a great amount of cryogenic energy, due to its temperature which is very low (110 K). The cryogenic energy can be extracted during the process of regasification. In other words, LNG can be used as an alternative energy supply. Nonetheless, the conventional regasification methods waste the cold energy and still need a great amount of energy supply [14,15,16,17,18]. Hence, investigation and development of cold recovery processes, has been the aim of many studies in the LNG regasification process. One of the most reliable methods for electricity power production is cold recovery process of LNG. About 16 cryogenic power plants using the recovered cryogenic energy of LNG have been built in Japan since 1979 to 2000 [19]. Efficiencies and performances of aforementioned cryogenic power plants have been continually enhanced. Dispenza [20,21] presented a modified process in a combined heat and power (CHP) system, which utilized LNG stream through the regasification stage, as a cold source. Szargut [22] evaluated the performance of three different cryogenic power plants. 

Several studies have focused on improving the performance of LNG cold energy recovery, by combining conventional power cycles or by advance optimization of the main variables of the cycles. Choi [23] presented a cascade Rankine cycle in order to recover LNG cold energy. The results showed that by increasing the stage numbers, exergy and energy efficiencies and total power output would enhance (<3 stages). Gomez [24] designed a new closed Brayton cycle power plant which includes a steam Rankine cycle, in which the regasification of LNG provided the available cold exergy. Still Gomez [14], evaluated the traditional thermodynamic cycles in order to enhance the power plant performance by using the cryogenic energy of LNG and provided a selection criterion for the working fluids. Dong [25] studied and modified a model, which used the LNG cryogenic energy to generate power by Stirling cycle.

The properties of working fluids and operational conditions, directly affect the power generation efficiency. Accordingly, some researchers studied the working fluid properties to enhance [26] the recovered cold energy of LNG. CO_2_ is a proper choice for power generation systems by employing LNG cold energy [27,28]. It is shown that low-temperature natural gas can be employed as a heat sink to condense the CO_2_. Additionally, employing CO_2_ can be considered as a solution for some environmental issues including rising sea levels and global warming. Some researchers have investigated capturing CO_2_ over cold energy recovery in LNG-fueled power plants. Liu [29] performed a thermo-economic analysis on a zero-CO_2_ emission and refrigeration cogeneration cycle. Zhang [30] studied a new configuration of the LNG-fueled power plant that liquefies the CO_2_ after combustion stage, and captures it without additional energy use. Alabdulkarem [31] studied an LNG plant with CO_2_ capture and sequestration in order to decrease the energy use through the CO_2_ liquefaction process. These investigations were subjected to ‘self-capturing’ which is related to the post-capture system. Nonetheless, some industries such as magnetite processing generate large amounts of CO_2_ without combustion.

One of the promising approaches with low emission and high efficiency in comparison with fossil fuel-based systems is Solid oxide fuel cell (SOFC) [32]. SOFC typically performs at a high temperature, approximately 600–1000 °C [33]. This technology has the ability to be integrated with other classical and regular thermodynamic cycles for enhancing the energy conversion efficiency. SOFC-GT system is a general approach to recover the waste heat from the SOFC. This system is provided by integrating a SOFC and a gas turbine (GT) as the bottoming cycle to improve the total efficiency via recovering waste heat from SOFC output. The theoretical backgrounds and conceptual models of the hybrid SOFC-GT system were studied and analyzed by various scholars for decades [34,35,36,37,38,39,40,41,42,43,44,45,46,47]. 

Solid oxide fuel cells have the ability to be coupled with bottoming power cycles, including Brayton [48], Rankine [49], Kalina [50] and Stirling [51] cycles, tri-generation systems [52], and renewable energy systems [53], to reach efficient power generation up to 80% efficiency [54,55].

Integrating SOFCs with high output temperatures (i.e., up to 1000 °C), with a bottoming steam Rankine cycle (SRC) [56,57,58] or organic Rankine cycle (ORC) [59,60,61,62,63,64], might limit SOFC operation temperature, and/or increase Rankine working fluid critical temperature. In spite of reported energy efficiencies of up to 71% [57], studies of SOFC-SRCs are limited. A single pressure level superheated SRC with hybrid SOFC-SRC thermal efficiencies of 62–68% has been used by Rokhi, depending cycle structure and operating conditions [63,64]. Gandiglio et al. [49] reported thermal efficiency of 65% by employing a three-pressure level SRC for recovering the waste heat of SOFC. Mehrpooya et al. [58] coupled a SOFC with a three-pressure level regenerative SRC at live steam pressures of 60–100 bar in order to reach 62.4% of electrical efficiency. ORCs have lower operating pressure, more compact layout and simpler structure, enhanced reliability, and reduced maintenance in comparison with SRCs [65].

Generally, SOFC is supplied by the methane as fuel. The methane is supported by the natural gas that is usually accumulated as LNG at −161 °C for large scale of manufacturing use. Throughout the natural gas liquefying process, a huge amount of mechanical energy is employed in the refrigeration process. Consequently, LNG comprises considerable cold energy. If the LNG cold energy could be retrieved in the fuel feeding process for SOFC, the efficiency of the suggested SOFC-GT-ORC unified power system could be increased. Various methods of LNG cold energy employing were proposed in previous years [66,67,68,69,70] including material freezing, intake air-cooling, and power generation. Liu and Guo [71] suggested an innovative cryogenic cycle via employing working fluids, comprising a binary mixture and improving the energy retrieval effectiveness of a LNG cold power creation, integrating with a vapor absorption process. The outcomes of simulation revealed that the innovative power cycle with LNG had significantly higher efficiency than the conventional ORC. Song et al. [27] suggested a transcritical CO_2_ power system powered by solar energy via employing LNG as heat sink. Their study revealed that the entire power cycle efficiency increased by employing the LNG cold energy underneath the particular situation and it can be additionally raised via parametric optimization.

Based on the literature review, several studies have been conducted on integrating various cycles with SOFC; however, there few studies focused on SOFC-GT-ORC. For instance, Eveloy et al. [72] investigated SOFC-GT-ORC systems and observed an enhancement in power generation by integrating these systems. Utilizing SOFC-GT-ORC for micro-scale power generation was investigated by Ebrahimi et al. [73]. It was concluded that these types of systems are efficient for power generation. In the present study, in addition to the mentioned configuration, LNG cold energy is utilized in order to achieve higher efficiency. Using LNG as a heat sink in SOFC-GT-ORC system was investigated by Yan et al. [74]; however, exergy analysis was not performed by the authors which is conducted in this study. Exergy analysis enables designers to distinguish the components with high exergy destruction rate and helps them to optimize the system more appropriately. The proposed system can be used for small-scale power generation by heat recovery from SOFCs utilized in industrial activities which consume LNG as fuel. Integrating the components (SOFC, ORC, GT and LNG as a heat sink) will lead to a high efficiency of the overall system. 

In the suggested SOFC-GT-ORC unified power system, LNG is employed as a heat sink to cool the compressor inlets, to condense the turbine outlet and lastly supply the fuel for the SOFC. In order to improve the power output of the ORC, LNG can decrease the temperature of condensing to a very low value, owing to considerable decreasing in the back pressure of turbine via the cryogenic condenser. In addition, LNG can turn cold the air in the intercooler between the two compressors that decreases the compressor intake air temperature, results in lower required work for the compression process.

In this paper, an integrated power system is studied by applying exergy and energy analyses as the principal methods. First, the integrated power system is described; afterward, the primary concepts of the simulation are discussed. Subsequently, results of the simulation, exergy analysis, and composite curves of heat exchangers are represented and discussed. In this design investigated of all components by Pinch technology and Exergy analysis. these tools for this design system used the first time. 

## 2. System Description

**Figure 1:**
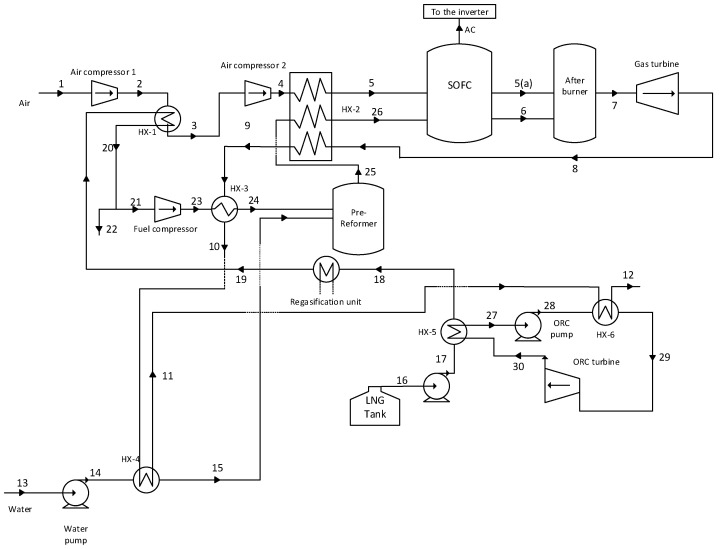
Flowsheet of the integrated power generation system including SOFC-GT-ORC with LNG as heat sink.

The flow sheet of an integrated power generation system, which uses LNG as a heat sink, is shown in Figure 1. In Figure 1, two compressors are used to compress the air stream to the SOFC stack operating pressure. The outlet air stream of the compressor (air compressor 1) is cooled in the heat exchanger (HX-1) by the low-temperature natural gas stream. Subsequently, after compression by the compressor (air compressor 2), the air stream is heated in the heat exchanger (HX-2) by the outlet stream of the gas turbine. The water stream (13) is pumped to the pre-reformer operating pressure and heated in the heat exchanger (HX-4) by the exhaust of the heat exchanger (HX-3) before entering to the pre-reformer reactor as steam. 

On the other hand, the LNG stream after heating in several heat exchangers (HX-5, HX-1, and refrigeration storage) is compressed to the pre-reformer operating pressure. Afterwards, the natural gas stream is heated in the heat exchanger (HX-3) by the outlet stream of the heat exchanger (HX-2) and enters to the pre-reforming reactor. In the pre-reformer reactor, regarding reforming reactions, a fraction of the natural gas is transformed into hydrogen. The product of the pre-reformer reactor (25), which contains the hydrogen heated in the heat exchanger (HX-2), enters to the SOFC stack as stream (26). The air stream, after preheating in the heat exchanger (HX-2) and reaching to an appropriate temperature, enters the SOFC stack, where electrochemical and reforming reactions happen concurrently. The reforming reaction is an endothermic one; therefore, a part of the heat emitted from the electrochemical reactions is consumed by the reforming reaction. A DC current is produced by the electrochemical reaction, which the inverter converts into AC.

The outlet streams of the anode and the cathode of the SOFC, (6) and (5(a)), respectively, enter the afterburner reactor. The untransformed part of the fuel in SOFC reactor burns in the afterburner. The outlet of the afterburner reactor with high pressure and the temperature is expanded by the gas turbine to produce power. As aforementioned, the exhaust of the gas turbine is used to heat up the air, natural gas, and water streams.

HCFC-123 is used as working fluid in the ORC Brayton cycle. The organic working fluid of the ORC cycle as stream (27) is compressed by the pump (ORC pump) after leaving the heat exchanger (HX-5) as a saturated liquid. Afterwards, the working fluid as stream 28 is introduced into the heat exchanger (HX-6) where it is heated and converted to the superheated vapor. The high-pressure superheat vapor (29) is expanded to low-pressure vapor by ORC turbine to produce power. The exhaust of the ORC turbine (30) is fed to the heat exchanger (HX-5) and is cooled by liquid natural gas to the saturated liquid.

HCFC-123 is used as working fluid in the ORC Brayton cycle. The organic working fluid of the ORC cycle as stream (27) is compressed by the pump (ORC pump) after leaving the heat exchanger (HX-5) as a saturated liquid. Afterwards, the working fluid as stream (28) is introduced into the heat exchanger (HX-6) where it is heated and converted to the superheated vapor. The high-pressure superheat vapor (29) is expanded to low-pressure vapor by ORC turbine to produce power. The exhaust of the ORC turbine (30) is fed to the heat exchanger (HX-5) and is cooled by liquid natural gas to the saturated liquid. 

## 3. Mathematical Model

### 3.1. SOFC Model

In order to model the system, chemical and thermodynamic relationship between species and components of the system are utilized which are explained for each sub-system. Chemical energy of the fuel is transformed into the electricity in the SOFCs. Oxygen ions are created in the cathode and tracked to the anode via a membrane. Created electrons in the anode move in the cathode after passing over an outer circuit; consequently, electrical current is generated [75]. Electrochemical reactions in the cathode and anode are as below, correspondingly:

(1) $$2H_{2}+2O^{2-}\to 2H_{2}O+4e^{-}$$

(2) $$O_{2}+4e^{-}\to 2O^{2-}$$

Owing to restricted accessibility, the power generation’s requisite hydrogen in the fuel cells has to be taking out of the other fuel [76]. At present, fossil fuels including natural gas, coal, and oil are employed for hydrogen production. The supreme cost-effective approach of hydrogen production in large scales is improving the fossil fuels, where the most economical one is natural gas [77]. Relation 3 depicts an endothermic reaction for methane steam improving:

(3) $${\mathrm{CH}}_{4}{+H}_{2}O\to {\mathrm{CO}+3H}_{2}\begin{array}{cc} & \left(r_{1}\right) \end{array}$$

Additional hydrogen can be generated by the water gas shift reaction that is an exothermic reaction.

(4) $${\mathrm{CO}+H}_{2}O\to {\mathrm{CO}}_{2}{+H}_{2}\begin{array}{cc} & \left(r_{2}\right) \end{array}$$

Relation 5 can present the overall reaction.

(5) $${\mathrm{CH}}_{4}{+2H}_{2}O\to {\mathrm{CO}}_{2}{+4H}_{2}\begin{array}{cc} & \left(r_{3}\right) \end{array}$$

The fuel improvement can be performed inside the SOFC stack. Internal improvement is grouped into two categories: indirect and direct. Indirect contact is performed through a distinct reactor connected to the stack; however, improving and electrochemical reactions happen at the same time interior the SOFC stack on the anode in direct approach [78]. Direct internal improvement has several benefits in comparison with external improvement. To avoid abrupt temperature variations and its coking occurrences, a portion of the fuel improvement (20–30%) is achieved outside the stack [76].

#### 3.1.1. Pre-Reformer Reactions Kinetics 

Numerous kinetic models were proposed for WGS and SMR reactions. These kinetic reactions are functions of a type of catalyst and working conditions. WGS and SMR kinetics on an optimized Ni/a-Al_2_O_3_ catalyst, within broad ranges of pressure (120–600 kPa) and temperature (748–823 K) are studied [79]. Developed kinetic reactions are obtained founded on the Freundlich's adsorption model and Langmuir-Hinshelwood-Hougen-Watson (LH-HW) method. LH-HW kinetic rate formulations are used for proposing and model the heterogeneous catalytic reactors. According to this approach, the rate calculating stage is the reaction on the surface of the catalyst [80]. In order to develop the approach, assumptions including plug flow in the reactor, steady state process, insignificant pressure drop, isothermal situations predominate, and no intra-particle and interphase mass transfer restrictions are taken into account [79]. The following expressions formulate the reaction kinetics:

(6) $$r_{1}=\frac{5.922\times 10^{8}\exp(\frac{25162}{T})}{P_{H_{2}}^{1.25}}\times (\frac{P_{CH_{4}}\times P_{H_{2}O}^{0.5}-\frac{P_{CO}P_{H_{2}}^{3}}{1.198\times 10^{17}\exp(-\frac{26830}{T})\times P_{H_{2}O}^{0.5}}}{{\left(DEN\right)}^{2}})$$

(7) $$r_{2}=\frac{6.028\times 10^{-4}\exp(\frac{1852}{T})}{P_{H_{2}}^{0.5}}\times (\frac{P_{CO}\times P_{H_{2}O}^{0.5}-\frac{P_{CO}P_{H_{2}}^{1}}{1.767\times 10^{-2}\exp(\frac{4400}{T})\times P_{H_{2}O}^{0.5}}}{{\left(DEN\right)}^{2}})$$

(8) $$r_{3}=\frac{1.093\times 10^{3}\exp(\frac{13158}{T})}{P_{H_{2}}^{1.75}}\times (\frac{P_{CH_{4}}\times P_{H_{2}O}^{1}-\frac{P_{CO_{2}}P_{H_{2}}^{4}}{2.117\times 10^{15}\exp(-\frac{22430}{T})\times P_{H_{2}O}^{1}}}{{\left(DEN\right)}^{2}})$$

(9) $$\begin{array}{l} DEN=1+(5.127\times 10^{-13}\exp(\frac{13158}{T}))P_{CO}+5.68\times 10^{-10}\exp(\frac{11234}{T})P_{H_{2}}^{0.5}\\ \;+9.251\exp(\frac{-1912}{T})\frac{P_{H_{2}O}}{P_{H_{2}}} \end{array}$$

In which *P_i_* (kPa) stands for the species *i* partial pressure and *T* (K) represents the temperature of the reactor. The units for *r*_1_, *r*_2_, and *r*_3_ are kmol/s·kg·cat. Owing to the unit change from kmol/s·kg·cat to kmol/s·m^3^, the bed density for this reactor is 1780 kg·cat/m^3^.

#### 3.1.2. Internal and Electrochemical Reforming Reactions Kinetics

For methane conversion on Ni/YSZ cermet, there are numerous kinetics. They are driven based on the various situations including working circumstance and material composition. The Achenbach and Riensche model is one of the reaction rates that have broadly been employed for modeling objects. This type of kinetic is an Arrhenius kinetic reaction rate:

(10) $$R_{r}=4274\times P_{CH_{4}}\times \exp(\frac{-9863}{T})\times A_{S}$$

In which, *P_CH_*_4_ stands for the methane partial pressure in the gas bulk, *A_s_* denotes the active surface area to the volume ratio and *T* represents the mean cell temperature. Steam to carbon ratio, pressure, and temperature range for this kinetic expression are correspondingly 2.6–8, 1.1–2.8 bar and 700–940 °C,. Their investigations are based on the anode geometric area with 1.4 mm anode cermet including 80 wt % ZrO_2_ and 20 wt % Ni. In the anode, WGS Kinetics reaction can be determined based on the Arrhenius approach:

(11) $$R_{s}=0.0171\times 10^{10}\times \exp(\frac{-12421}{T})\times (P_{H_{2}O}P_{CO}-\frac{P_{H_{2}}P_{CO_{2}}}{0.019\exp(\frac{4276}{T})})$$

Electrochemical *H*_2_ production rate can be formulated by the following Equation (12):

(12) $$R_{H_{2}}=\frac{-J}{2F}$$

In which *J* stands for the current density and *F* represents the Faraday constant. Along with hydrogen, CO could also be employed as fuel in SOFC, and its oxidation reaction can be presented as follows:

(13) $${\mathrm{CO}+O}^{2-}\to {\mathrm{CO}}_{2}{+2e}^{-}$$

When both H_2_ and CO are available at the same time, CO has a lower tendency to participate in the electrochemical reactions in comparison with reacting with water [56]. Similarly, the H_2_ oxidation rate is more than three times higher than the CO oxidation rate. For the conditions where the WGS reaction is in equilibrium, the Nernst potential created via H_2_ oxidation equals to CO oxidation [57,58]. Owing to the dominance of H_2_ oxidation under various circumstances, the influence of the CO oxidation has been ignored [59,60,61,62]. It is deduced that the cell performance is not influenced considerably by CO oxidation [63,66]. Outcomes of the experimental data depict that hydrogen oxidation is the principal anodic process in the SOFCs, whereas the shift equilibrium used CO [36].

#### 3.1.3. SOFC Voltage

The fuel cell real output voltage is calculated by subtracting the voltage losses from the thermodynamically ideal voltage [37]. The losses in the fuel cells occur due to irreversibilities [81]. In order to determine the output electricity, it is necessary to obtain an actual voltage which leads to more precise efficiency calculation. By applying Equation (14), the actual voltage is calculated in the simulation:

(14) $$V=E_{thermo}-\eta_{act}-\eta_{ohmic}-\eta_{conc}$$

*V* stands for the actual voltage, *η_act_* stands for the activation losses, *E_thermo_* represents the ideal voltage, *η_conc_* represents the concentration losses, and *η_ohmic_* denotes the Ohmic losses. The bulk mole fraction of components and the working temperature of fuel cell affect output ideal voltage. By considering the influential parameters on the output ideal voltage, it can be determined as follows:

(15) $$E_{thermo}=1.177-0.06855(t-1)-0.0165(t\ln t-t)-\frac{RT}{nF}\ln(\frac{P_{H_{2}O}^{0}}{P_{O_{2}}^{0}{}^{0.5}P_{H_{2}}^{0}})$$

(16) $$t=\frac{T}{298.15}$$

In which *T* stands for the mean cell temperature, and *P*^0^*_H_*_2*O*_; *P*^0^*_H_*_2_; *P*^0^*_O_*_2_ denote the bulk mole fraction of H_2_O, H_2_ and O_2_, respectively. As mentioned, it is necessary to subtract the losses from the ideal voltage to obtain the actual output voltage; therefore, each loss is calculated based on the effective factors. First of all, the losses due to electrical resistances are calculated. The resistance of the fuel cell elements (cathode, anode, interconnects and electrolyte) to the ion flow and electrons reduces the performance of the cell which is named Ohmic loss, $\eta_{ohmic}$ [35]. It can be determined as follows:

(17) $$\eta_{ohmic}=j\times \sum_{i}{\left(\sigma_{i}\right)}^{-1}\times \delta_{i}$$

σ stands for the electronic or ionic conductivity and $\delta_{i}$ represents the cathode, anode, interconnector, and electrolyte thickness. Another influential loss is related to the activation energy required for the electrochemical reactions [82]; this type of loss is related to the mechanism of the reactions which happen across the electrodes and [83]. The rate at which reactants are transformed into the products is restricted due to the required activation energy. Consequently, a portion of the energy is used owing to electrochemical reactions at the cathode and anode. This kind of loss is named activation loss, *η_act_* [37]. Various parameters affecting the activation loss must be taken into consideration in determining this loss. The equation below expresses the relation between the activation loss and current density:

(18) $$j=j_{0}\left[\exp(\alpha \frac{n_{e}F}{RT}\eta_{act})-\exp(-(1-\alpha )\frac{n_{e}F}{RT}\eta_{act})\right]$$

where *n_e_* stands for the number of electrons transmitted per electrochemical reaction, *a* represents the transfer coefficient and *j*_0_ denotes the exchange current density of the cathode and anode that is determined by the following equations [64]:

(19) $$j_{0,an}=\gamma_{an}\frac{RT}{n_{e}F}\exp(-\frac{E_{act,an}}{RT})$$

(20) $$j_{0,\mathrm{cat}}=\gamma_{cat}\frac{RT}{n_{e}F}\exp(-\frac{E_{act,cat}}{RT})$$

By assuming *a* = 0.5 [84], activation loss is determined as follows:

(21) $$\eta_{act}=\frac{2RT}{n_{e}F}\sinh^{-1}(\frac{j}{2j_{0,cat}})+\frac{2RT}{n_{e}F}\sinh^{-1}(\frac{j}{2j_{0,an}})$$

Another important loss in SOFCs is the concentration loss (*η_conc_*) which is due to concentration gradient at the surface of the electrodes. The porous structure of the electrodes is one of the significant factors which results in the reaction sites have dissimilar concentration compared with the bulk [35]. Concentration losses depend on various factors such as working temperature, bulk and site partial pressure. Concentration loss is determined by the equation below:

(22) $$\eta_{conc}=\frac{RT}{n_{e}F}\ln(\frac{P_{H_{2}O}^{*}P_{H_{2}}^{0}}{P_{H_{2}O}^{0}P_{H_{2}}^{*}})+\frac{RT}{2n_{e}F}ln(\frac{P_{O_{2}}^{0}}{P_{O_{2}}^{*}})$$

where, *P_oi_* and *P_i_* represent reaction bulk and sites partial pressure of the species, correspondingly. *P_i_* is calculated as follows:

(23) $$P_{H_{2}}^{*}=P_{H_{2}}^{0}-\frac{j\times \delta_{an}\times R\times T}{n_{e}FD_{H_{2}}^{eff}}$$

(24) $$P_{H_{2}O}^{*}=P_{H_{2}O}^{0}-\frac{j\times \delta_{an}\times R\times T}{n_{e}FD_{H_{2}O}^{eff}}$$

(25) $$P_{O_{2}}^{*}=P_{O_{2}}^{0}-\frac{j\times \delta_{cat}\times R\times T}{n_{e}FD_{O_{2}}^{eff}}$$

in which, *P* stands for the total pressure, and *D^eff^_i_* denotes the effective diffusion coefficient of the species *i* at the cathode and anode. In porous media, diffusion is calculated via the Knudsen diffusion and the binary molecular diffusion [84]:

(26) $$D_{i}^{eff}=\frac{\varepsilon }{\tau }{\left(\frac{1}{D_{i,M}}+\frac{1}{D_{i,K}}\right)}^{-1}$$

where *D_i_*_,*K*_ represents the Knudsen diffusion, *D_i_*_,*M*_ stands for the diffusivity of species *i* in the multi-component gas mixture, *t* and *ε* represent the tortuosity and porosity of the materials, correspondingly. Knudsen diffusion depends on cell temperature, the diameter of structure pore, and molecular weights of components [84]. Knudsen diffusion is determined by the following equation:

(27) $$D_{i,K}=4850\times d_{pore}\sqrt{\frac{T}{M_{i}}}$$

where *T* represents the mean cell temperature, $d_{pore}$ stands for the structure pore diameter, and $M_{i}$ denotes the molecular weight of species *i*. The binary diffusion coefficient between species *j* and *i* in the free space is calculated via the Chapmane Enskog equation:

(28) $$D_{ij}=0.0018583\frac{T^{\frac{3}{2}}\times M_{ij}^{0.5}}{P\times {\tilde{\sigma }}_{ij}^{2}\times \Omega_{D}}$$

$M_{ij}$ denotes average molecular weight, $\Omega_{D}$ represents the collision diffusion integral based on the Lennarde Jones potential, and $\tilde{\sigma }$ stands for the collision diameter (Lennard Jones length):

(29) $$M_{ij}={\left(\frac{1}{M_{i}}+\frac{1}{M_{j}}\right)}^{-1}$$

Diffusivity of species *i* in the multi-component gas mixture $D_{i,M}$ could be evaluated with Wilke’s equation:

(30) $$D_{i,M}=\frac{1-x_{i}}{\sum_{j\neq i}^{n}\frac{x_{j}}{D_{ij}}}$$

Finally, Equation (31) evaluates the electrical power that produced by a stack:

(31) $$Power_{stack}=N_{cells}\times A_{cell}\times j\times V$$

Meanwhile, all required assumptions for evaluation of the cell voltage are presented in Table 1, based on [36,64,85].

### 3.2. Energy Analysis 

The thermodynamic efficiency of the power cycle is defined as the ratio of net output power to the input heat absorbed by the working fluid. The mass and energy balances of any control volume at steady state with negligible kinetic and potential energy alterations are written as follows:

(32) $${\sum \dot{m}}_{i}={\sum \dot{m}}_{e}$$

(33) $$\dot{Q}-\dot{W}=\sum \dot{m_{e}}H_{e}-{\sum \dot{m}}_{i}H_{i}$$

Based on the above equations, energy balance equation for the SOFC defined as [81]:

(34) $$\Sigma {\dot{m}}_{in}H_{in}+{\dot{m}}_{fuel}Q_{fuel,LHV}=\Sigma {\dot{m}}_{Out}H_{Out}+W_{SOFC}$$

The first law efficiency for a power generation cycle is as follows:

(35) $$\eta =\frac{\dot{W_{net}}}{\dot{Q_{input}}}$$

### 3.3. Exergy Analysis

Exergy analysis is applied to the integrated power system to determine irreversibilities at the various operating stages. The dead state (reference point) for all exergy evaluations is adjusted to *T_dead-state_* = 298.15 K and *P_dead-state_* = 101.3 kPa. 

The exergy is defined as the quality of various types of energy in relation to a given system. The exergy analysis is a relatively new method based on the concepts of both the first and second laws of thermodynamics. An exergy analysis applied to a process can indicate how much of the usable work potential, or exergy, introduced as the input to the process is destructed.

The concept of the theorem employed is based on the approach described in [87]. Exergy of a system can be divided into four parts including chemical, physical, potential and kinetic [88]. Since the kinetic and potential exergies are insignificance, these types are neglected [88]. As a consequence, the total exergy of the streams can be determined by Equation (36) in which the total exergy rate (${\dot{Ex}}_{tot}$) is calculated by summation of the chemical exergy rate (${\dot{Ex}}_{ch}$).

(36) $${\dot{Ex}}_{tot}={\dot{Ex}}_{ph}+{\dot{Ex}}_{ch}$$

In which the physical exergy rate can be determined by Equation (37) [89].

(37) $${\dot{Ex}}_{ph}=({\dot{H}}_{T,P}-{\dot{H}}_{T_{{}^{0}},P_{{}^{0}}})-T_{{}^{0}}({\dot{S}}_{T,P}-{\dot{S}}_{T_{{}^{0}},P_{{}^{0}}})$$

where, ${\dot{S}}_{T,P}$ and ${\dot{H}}_{T,P}$, denote the entropy and enthalpy rates of the streams at working pressure and temperature *(P*, *T)*, correspondingly, while ${\dot{S}}_{T_{{}^{0}},P_{{}^{0}}}$ and ${\dot{H}}_{T_{{}^{0}},P_{{}^{0}}}$ represent the entropy and enthalpy rates at the ambient temperature and pressure (*P* = 1 atm, *T* = 298.15 K), respectively. In Equation (35), chemical exergy rate of the mixture can be determined by Equation (38) [85] in which *x_i_* and *Ė_i_*^0^, represent mole fraction of component *i*, and standard chemical exergy, correspondingly:

(38) $${\dot{Ex}}_{ch}=\sum x_{i}{\dot{E}}_{i}^{0}+\dot{G}-\sum x_{i}{\dot{G}}_{i}$$

Exergy balance for any control volume at the steady state condition with negligible kinetic and potential exergy alterations is as follows [87]:

(39) $${\dot{Ex}}_{heat}-\dot{W}=\dot{Ex_{tot,in}}-\dot{Ex_{tot,out}}+\dot{I}$$

where $\dot{I}$ stands for exergy destruction rate and ${\dot{Ex}}_{heat}$ shows the net exergy transfers by heat at temperature *T* which can be obtained by [87]:

(40) $${\dot{Ex}}_{heat}=(1-\frac{T_{0}}{T})\dot{Q}$$

The exergy efficiency of a unit or plant can be calculated by the ratio of produced exergy to input exergy. Nevertheless, rational exergy, as an exergy-based efficiency, can be applied to have much more logical analysis for a plant due to high accuracy in determining input exergy to a plant. The rational exergy can be determined as [87]:

(41) $$\mathrm{Rational}\;\mathrm{efficiency}=\frac{\mathrm{Actual}\;\mathrm{exergy}\;\mathrm{output}}{\mathrm{Actual}\;\mathrm{exergy}\;\mathrm{input}}$$

In Equation (40), the term actual exergy input represents the net exergy that the plant has access to it or is available for it. In another word, the plant has the equipment to benefit from these values of exergy. Summary of the relationships and the definitions that are used to calculate the exergy destruction and exergy efficiency of the principal components is shown in Table 2.

## 4. Process Simulation and Assumption

In this work, the Aspen-HYSYS commercial software is utilized to simulate the processes. Comprehensive thermodynamic databases of the Aspen-HYSYS software provide chemical and physical properties of heterogeneous mixtures at variegated operating pressures and temperatures with a single fluid package. This chemical process simulator makes the simulation of the complex processes with lots of streams possible. Anderson et al. [90] performed a simulation of the SOFC reactor by Aspen-HYSYS. The intrinsic features of the Aspen-HYSYS without any linked codes formed their model of the simulation. They showed that the results of their model are reasonable over a wide range of conditions. Mehrpooya et al. [58] performed a study on a combined system containing SOFC, gas turbine, ammonia-water absorption refrigeration system and Rankine steam cycle. The SOFC part of their system was simulated by Aspen-HYSYS, and they showed that the electrochemical approach could estimate the experimental data with high precision. The assumptions for the simulation are shown in Table 3 [74]. 

These assumptions include the SOFC model input data and the efficiency of the rotary instruments of the proposed system. These assumptions are taken based on a study that is carried out by Yan et al. [74]. 

The composition of the liquid natural gas stream which is utilized in the process is shown in Table 4, which is assumed based on operating LNG plant products. Soave-Redlich-Kwong (SRK) [91] and Peng-Robinson (PR) [86] equation of states are appropriate for the determination of thermodynamic properties of the operating conditions and the composition of the streams in the SOFC system. Therefore, the Peng-Robinson (PR) equation of states employed as a proper fluid package for the calculation of the thermodynamic properties.

## 5. Results and Discussion

### 5.1. Exergy Analysis

In this section, the results of the simulation are shown and the exergy and energy analyses of the system are carried out. 

**Figure 2:**
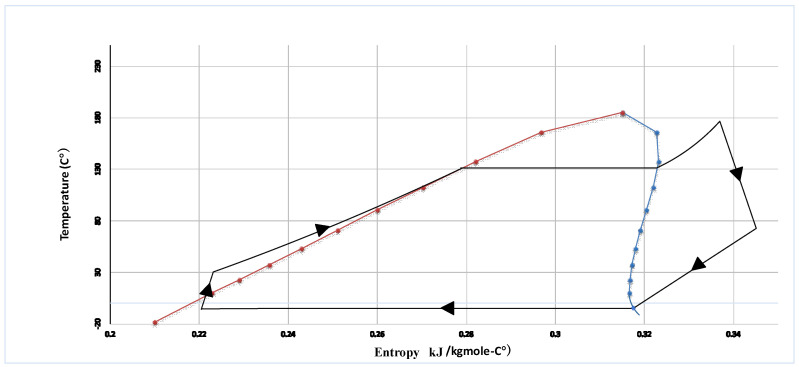
T-S diagram of the organic Rankine cycle.

Table 5 represents the thermodynamic properties of the integrated power system such as pressure, molar flow rate of each stream, and temperature. Table 6 indicates the performance of the integrated power system. Additionally, the chemical, physical and total exergy of each stream is represented in Table 5. As Table 6 shows, the calculated energy efficiency of the organic Rankine cycle (21.93%) is low compared to the modern power plants and it shows that a great deal of energy is dissipated by the considered cycle. However, efficiencies founded on energy can be vague and ambiguous, since it does not consider the quality of the energy. In fact, losses can enjoy a high amount of energy while in terms of thermodynamic, they are not significance and appreciable owing to their low quality. Nevertheless, efficiencies based on exergy can determine the quality of energy and measure of the deviation from ideality. According to Figure 2, showing the T-S diagram of the organic Rankine cycle, it could be considered that this cycle operates at low temperatures. The exergy analysis’ results reveal that the exergy efficiency associated with the organic Rankine cycle is 64.92%, which is high enough due to its low operating temperatures and low quality of the energy at these low temperatures. 

**Figure 3:**
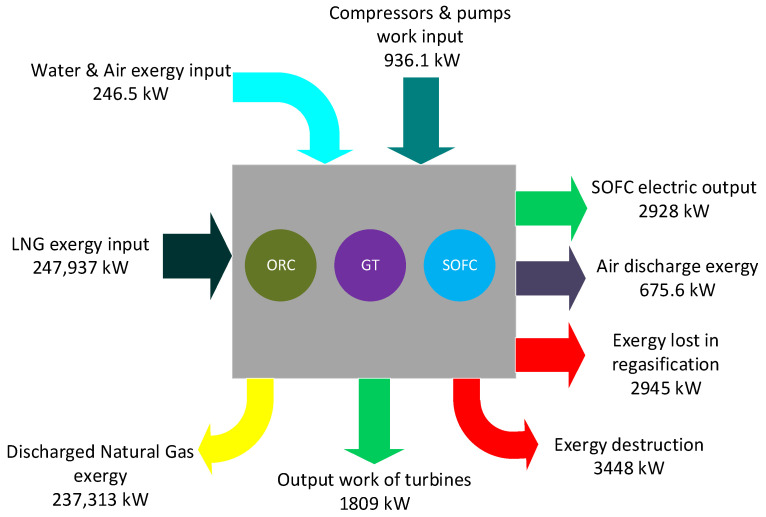
Various exergy input and output factors in the integrated power system.

Again, the exergy efficiency of the whole plant is measured to be 39.91% while the rational efficiency of the integrated power system is 53.46%. The reason for such a difference lies in a significant amount of exergy loss rate at the regasification unit due to the lack of appropriate equipment to benefit from this exergy. The rational efficiency indicates that over half of the available exergy inputs to the plant is transferred to the power and a proportion of the irreversibilities are inevitable owing to the physical, technological, and economic constraints. Figure 3 depicts diverse input and output parameters in the integrated power system. 

Again, the exergy efficiency of the whole plant is measured to be 39.91% while the rational efficiency of the integrated power system is 53.46%. The reason for such a difference lies in a significant amount of exergy loss rate at regasification unit due to the lack of appropriate equipment to benefit from these amounts of exergy.

The rational efficiency indicates that over half of the available exergy inputs to the plant is transferred to the power and a proportion of the irreversibilities are inevitable owing to the physical, technological, and economic constraints. Figure 3 depicts diverse input and output parameters in the integrated power system.

Furthermore, the exergy efficiency and exergy destruction of the influential components of the process are listed in Table 7. Regarding Table 7, the lowest exergy efficiency among components belongs to LNG pump due to the low temperature of the LNG stream and rough operating climate of this component. Furthermore, the exergy destruction rate of the afterburner is dominant over all other irreversibilities. This high exergy destruction rate indicates that appreciable opportunities for improvements exist in the afterburner rather than other equipment. In addition, the majority of the exergy efficiencies of the multi-stream heat exchangers are lower than 80% which show that the performance of the heat exchangers is far from lagging behind the ideality. 

**Figure 4:**
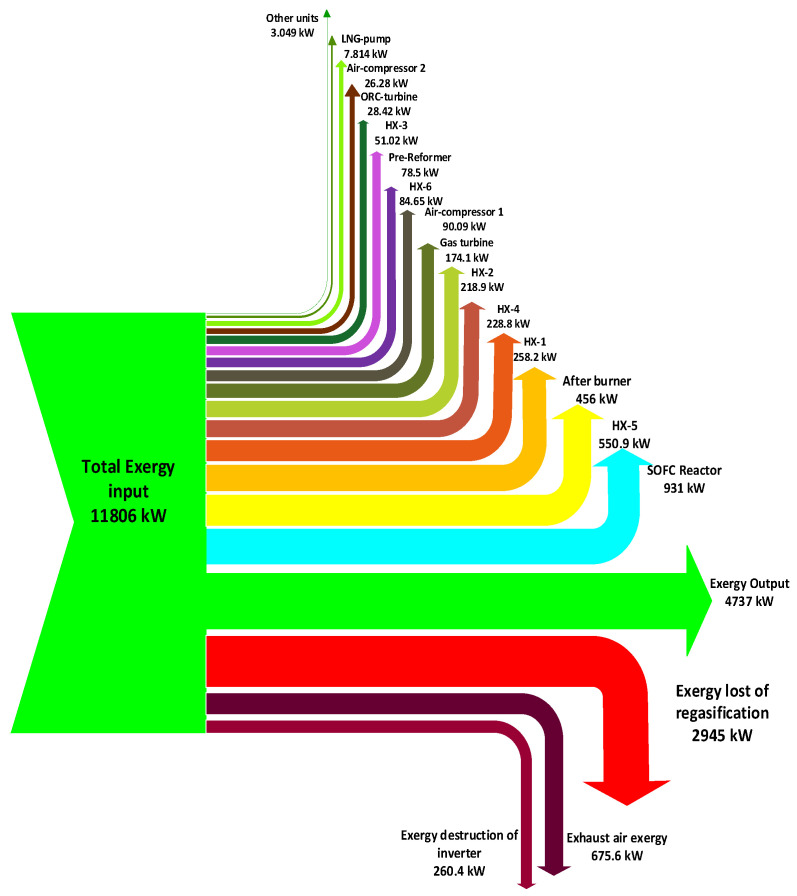
Exergy flow diagram depicting the exergy destruction in the integrated power plant.

According to Table 7, the heat exchanger (HX-5) that operates with LNG low-temperature stream, has the lowest exergy efficiency among other heat exchangers. The reason for this phenomenon is that, at lower temperatures with a constant rate of heat transfer, entropy generation has a higher value [92]. Figure 4 shows the various types of exergy destruction in the integrated power system as a Grassmann diagram. According to Figure 4 and Table 7, the SOFC reactor is ranked third in exergy destruction rate among other units despite its acceptable exergy efficiency compared to other equipment.

### 5.2. Composite Curves

A number of authors have described and applied composite curves to the heat exchangers of power plants. Klemes [93] and Bandyopadhyay [94] carried out studies about the importance of the composite curves in waste reduction in the heat exchangers. The composite curves (T-H diagrams) indicate the variation of the temperature in terms of the enthalpy contribution. In other words, by drawing the rates of enthalpy cumulatively against corresponding temperatures, one curve for the hot streams and one curve for the cold streams can be obtained. In these curves, the cooled curve (the hot composite curve) is always above the heated curve (the cold composite curve). By overlapping the interval between the composite curves, the corresponding heat exchanger can operate more efficiently. This overlapping can be achieved by moving the curves closer together horizontally. Economically, minimum approach temperature is equal to the point where the vertical distance between the curves has the smallest value while in terms of the thermodynamics, minimum approach temperature is the point where this distance becomes zero.

**Figure 5:**
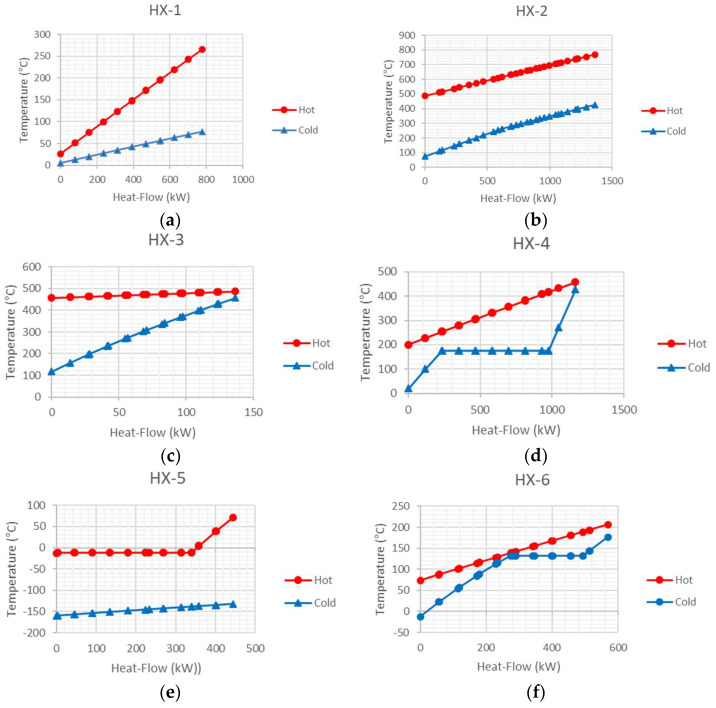
Hot (red) and cold (blue) composite curves for the heat exchangers.

The heat exchangers’ composite curves associated with the plant are depicted in Figure 5. Regarding Figure 5, at the heat exchanger (HX-1), the min approach temperature is near to 21.4 °C, and it is located just after the cold inlet side of the heat exchanger. The cold and hot pinch temperatures are 26.4 °C and 5 °C, respectively. On the other hand, the minimum approach temperature of the heat exchanger (HX-2) is near to 321.8 °C, which is located at the cold end of the heat exchanger.

As mentioned above, a high minimum approach temperature indicates that there is a great potential for optimizing the heat exchanger performance. However, economic limitations and sizing issues force one to choose a high minimum approach temperature. The cold composite curve of the heat exchanger (HX-4) shows that the curve appears horizontal at temperature 175 °C, due to the phase changing of the stream. The minimum approach temperature of this heat exchanger is located at the cold end of it and its value is approximately 36 °C. According to Figure 5, at the heat exchanger (HX-5), the min approach temperature is located just after the working fluid of the ORC cycle starts to condense. Due to the cryogenic temperatures of the LNG stream, the value of the minimum approach temperature is estimated as high as 127.5 °C. 

## 6. Conclusions

An integrated power generation system including the SOFC reactor, gas turbine, and organic Rankine cycle with LNG as a heat sink is simulated by the Aspen-HYSYS simulator, followed by energy and exergy analyses based on the results of the simulation. As the result of the energy analysis shown, the energy efficiency of the organic Rankine cycle (21.93%) is lower than other modern power plants despite its high exergy efficiency (64.92%). This fact shows the weakness of the energy efficiency in the determination of the quality of energy at low temperatures and significance of the efficient performance of organic Rankine cycle at these low temperatures. In addition, the exergy analysis illustrates that the exergy and rational efficiencies of the whole plant measured to be 39.91% and 53.46%, respectively. The most significant results of the exergy analysis are as follows:

(a) The exergy losses and difference between the rational and exergy efficiencies can be enumerated as the circumstances of low efficiencies of equipment in regasification unit.
(b) The highest values of exergy destruction rate associate with the afterburner and heat exchanger (HX-5), which are equal to 456 and 550.9 kW, respectively, indicating that appreciable potential for improvements exists in these units. The lowest value for exergy destruction belongs to water-pump which is equal to 0.0599 kW.
(c) It is worth to note that the high exergy destruction rate of the heat exchanger (HX-5) is due partially to the cryogenic temperature of the LNG stream, causing more entropy generation.
(d) The lowest exergy efficiency among components belongs to the LNG pump which is equal to 11.73%, owning to the rough operating climates of this unit.
(e) Due to the low exergy efficiency of the majority of the heat exchangers, there is a lack of heat recovery in the heat exchangers. The composite curves of the heat exchangers show that the minimum approach temperatures of some heat exchangers (HX-2 and HX-5) have high values, revealing that there is a great potential for optimizing the heat exchanger performance.
(f) Economical and technical restrictions and sizing matters dictate some exergy destruction rates to the heat exchangers and the whole plants, known as unavoidable irreversibilities.
(g) An exergy flow diagram is represented in order to get better insight into exergy destruction in different components of the system.

## Figures and Tables

**Table 1:** SOFC simulation assumptions.

Factor	Value
Cathode [86]	
Thickness (μm)	30
Average pore radius (μm)	0.5
Average particle diameter (μm)	2.5
Porosity	0.48
Tortuosity	5.4
Charge transfer coefficient	0.5
Anode [86]	
Thickness (μm)	750
Average pore radius (μm)	0.5
Average particle diameter (μm)	2.5
Specific area (m^−1^)	$1.025\times 10^{5}$
Porosity	0.35
Tortuosity	3.8
Charge transfer coefficient	0.5
Electrolyte [86]	
Thickness (μm)	25
Interconnect	
Thickness (μm)	1500
Parameters for exchange current density [71]	
Pre-exponential factor for cathode (A·cm^−2^)	$6.5\times 10^{7}$
Activation energy for cathode (kj·mole^−1^)	140
Pre-exponential factor for anode (A·cm^−2^)	$2.35\times 10^{7}$
Activation energy for anode (kj·mole^−1^)	137
Conductivity parameter (Ω^−1^·cm^−1^) [43]	
Anode	($95\times 10^{4}$/T) exp(−1150/T)
Cathode	($42\times 10^{4}$/T) exp(−1200/T)
Electrolyte	334 exp(−10,300/T)
Interconnect	($9.3\times 10^{4}$/T) exp(−1100/T)

**Table 2:** Summary of the relationships and the definitions used to calculate the exergy efficiency.

Components	Exergy Destruction	Exergy Efficiency
Compressors	$I=Ex_{i}-Ex_{0}=\sum {\left(\dot{m}.e\right)}_{i}+W-\;\sum {\left(\dot{m}.e\right)}_{0}$	$\varepsilon =\frac{\sum {\left(\dot{m}.e\right)}_{i}-\sum {\left(\dot{m}.e\right)}_{0}}{W}$
expanders	$I=Ex_{i}-Ex_{0}=\sum {\left(\dot{m}.e\right)}_{i}-W-\;\sum {\left(\dot{m}.e\right)}_{0}$	$\varepsilon =\frac{W}{\sum {\left(\dot{m}.e\right)}_{i}-\sum {\left(\dot{m}.e\right)}_{0}}$
heat exchangers	$I=Ex_{i}-Ex_{0}=\sum {\left(\dot{m}.e\right)}_{i}-\;\sum {\left(\dot{m}.e\right)}_{0}$	$\varepsilon =1-\left[{\left\{\frac{\sum \left(\dot{m}.\Delta e\right)}{\sum \left(\dot{m}.\Delta h\right)}\right\}}_{h}-{\left\{\frac{\sum \left(\dot{m}.\Delta e\right)}{\sum \left(\dot{m}.\Delta h\right)}\right\}}_{c}\right]$
pumps	$I=Ex_{i}-Ex_{0}=\sum {\left(\dot{m}.e\right)}_{i}+W-\;\sum {\left(\dot{m}.e\right)}_{0}$	$\varepsilon =\frac{\sum {\left(\dot{m}.e\right)}_{i}-\sum {\left(\dot{m}.e\right)}_{0}}{W}$
separators, drums and reformers	$I=Ex_{i}-Ex_{0}=\sum {\left(\dot{m}.e\right)}_{i}-\;\sum {\left(\dot{m}.e\right)}_{0}$	$\varepsilon =\frac{\sum {\left(\dot{m}.e\right)}_{0}}{\sum {\left(\dot{m}.e\right)}_{i}}$
SOFC	$I=Ex_{i}-Ex_{0}=\sum {\left(\dot{m}.e\right)}_{i}-\;\sum {\left(\dot{m}.e\right)}_{0}$	$\varepsilon =\frac{W}{\sum {\left(\dot{m}.e\right)}_{i}-\sum {\left(\dot{m}.e\right)}_{0}}$
Cycle/process	Summation of irreversibility of all devices	$\varepsilon =1-\frac{\mathrm{Total}\;\mathrm{irreversibillity}\;\mathrm{of}\;\mathrm{cycle}}{\mathrm{Total}\;\mathrm{exergy}\;\mathrm{input}\;\mathrm{to}\;\mathrm{cycle}}$

**Table 3:** Assumptions of the simulation for the integrated power system.

Parameter	Value
Ambient temperature (°C)	20 (293.15 K)
Ambient pressure (bar)	1.013
DC-AC inverter efficiency	90%
Pre-Reformer conversion	15%
Conversion in Combustor	100%
Minimum steam-to-carbon ratio	2.5
Fuel cell temperature (°C)	814 (1087 K)
SOFC operating pressure (bar)	8.890
Fuel utilization	0.85
Active surface area (cm^2^)	220
Number of cell	50,000
Exchange current density of anode (A/cm^2^)	0.6
Exchange current density of cathode (A/cm^2^)	0.22
Inlet temperature to the Pre-reforming (°C)	427 (700 K)
Inlet temperature to the SOFC (°C)	427 (700 K)
Thickness of the anode (cm)	0.01
Thickness of the cathode (cm)	0.22
Thickness of the interconnect (cm)	0.0085
Thickness of the electrolyte (cm)	0.004
Pressure ratio of the LNG pump	6
Pump efficiency	75%
ORC turbine efficiency	80%
Gas turbine efficiency	75%
Fuel compressor efficiency	82%
Air compressor efficiency	82%

**Table 4:** Composition of the liquid natural gas stream of the process.

Components	Mole Fraction
Methane	0.9800
Ethane	0.014
Propane	0.0040
n-Butane	0.0010
Nitrogen	0.0010

**Table 5:** Operating conditions for the process depicted in Figure 1.

Stream No.	Temperature (K)	Pressure (kPa)	Flow (kmol/h)	Physical Exergy (kW)	Chemical Exergy (kW)	Total Exergy (kW)
1	293.15	101.3	388.8	4.199	12.24	12.24
2	525.0	606.0	388.8	688.4	12.24	700.6
3	299.6	586.0	388.8	469.1	12.24	481.4
4	348.1	909.0	388.8	595.7	12.24	607.9
5	700.0	889.0	388.8	1071	12.24	1083
5(a)	1119	889.0	339.8	1789	21.74	1811
6	1119	889.0	522.9	1199	1661	2860
7	1352	889.0	489.6	3683	532.1	4215
8	1022	151.0	489.6	1855	532.1	2387
9	765.3	141.0	489.6	1032	532.1	1564
10	735.8	128.0	489.6	946.4	532.1	1479
11	479.2	118.0	489.6	337.2	532.1	869.3
12	347.5	104.3	489.6	143	532.1	675.6
13	293.1	101.0	72.00	0.0616	234.2	234.2
14	293.2	909.0	72.00	0.4033	234.2	234.6
15	700.0	899.0	72.00	381.4	234.2	615.5
16	113.1	121.0	1027	4880	243,057	247,937
17	113.4	726.0	1027	4881	243,057	247,938
18	140.7	726.0	1027	4281	243,057	247,342
19	278.1	676.0	1027	1340	243,057	244,397
20	345.9	606.0	1027	1301	243,057	244,358
21	346.0	606.0	28.80	36.49	6692	6729
22	346.0	606.0	998.2	1265	236,048	237,313
23	384.5	909.0	28.80	46.84	6692	6739
24	700.0	899.0	28.80	109.6	6692	6802
25	633.5	899.0	107.6	334.1	7005	7339
26	700.0	889.0	107.6	374.3	7005	7379
27	260.0	20.00	489.6	4.255	-	4.255
28	260.8	1500	44.26	5.848	-	5.848
29	450.0	1500	44.26	114.9	-	114.9
30	345.1	20.00	44.26	−40.80	-	−40.80

**Table 6:** Results of energy and exergy analyses of the integrated power system simulation.

Parameter	Value
SOFC operating temperature (°C)	845.9
After-burner combustion temperature (°C)	1057
ORC turbine inlet temperature (°C)	176.9
SOFC operating current density (A/m^2^)	4628
Cell operating voltage (V)	0.575
SOFC electrical power (kW)	2928
Air Compressor 1 power (kW)	759.5
Air Compressor 2 power (kW)	153.1
Fuel compressor power (kW)	11.89
ORC-Pump	2.383
LNG-Pump	8.807
Water-Pump	0.3839
Gas turbine power	1682
ORC turbine power	127.3
Energy efficiency of ORC (%)	21.93
Exergy efficiency of ORC (%)	64.92
Overall exergy efficiency of plant (%)	39.91
Rational efficiency of plant (%)	53.46

**Table 7:** Results of exergy destruction and exergy efficiency.

Components	Exergy Destruction (kW)	Exergy Efficiency (%)
Air-compressor 1	90.09	88.43
Air-compressor 2	26.28	82.83
Methane-compressor	2.253	81.63
Water-pump	0.0599	83.36
ORC-pump	0.7361	51.47
LNG-pump	7.814	11.73
Gas-turbine	174.1	89.48
ORC-turbine	28.42	77.69
HX-1	258.2	66.82
HX-2	218.9	76.94
HX-3	51.02	62.37
HX-4	228.8	80.35
HX-5	550.9	61.62
HX-6	84.65	85.05
Pre-Reforming	78.5	98.95
SOFC reactor	931	75.68
Inverter	260.4	91.83
After burner	456	90.23
